# Study on the Interaction between Isatin-β-Thiosemicarbazone and Calf Thymus DNA by Spectroscopic Techniques

**Published:** 2015

**Authors:** Parvaneh Pakravan, Shahla Masoudian

**Affiliations:** a Department of Chemistry, Zanjan Branch, Islamic Azad University, Zanjan, Iran.; b Department of Chemistry, Payame Noor University, I.R. of Iran.

**Keywords:** CT-DNA, Isatin-*β*-thiosemicarbazone, Neutral Red dye, Intercalation

## Abstract

The interaction between isatin-*β*-thiosemicarbazone (IBT) and calf thymus DNA (CT-DNA) was investigated in physiological buffer (pH 7.4) using Neutral Red (NR) dye as a spectral probe by UV–Vis absorption and ﬂuorescence spectroscopy, as well as viscosity measurements. The IBT is stabilized by intercalation in the DNA (K _[IBT –DNA]_ = 1.03×10^5^ M^−1^), and displaces the NR dye from the NR–DNA complex. The binding constants K_f_ and number of binding sites (n≈1) of IBT with DNA were obtained by fluorescence quenching method at different temperatures. Furthermore, the enthalpy and entropy of the reaction between IBT and CT-DNA showed that the reaction is enthalpy-favored and entropy-disfavored. The changes in the base stacking of CT-DNA upon the binding of IBT are reflected in the circular dichroic (CD) spectral studies. The viscosity increase of CT-DNA solution is another evidence to indicate that, IBT is able to be intercalated in the DNA base pairs.

## Introduction

**Figure 1:**
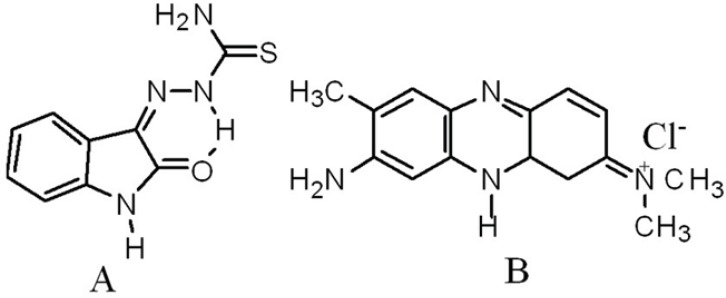
Structures of Isatin-*β*-thiosemicarbazone (IBT, A) and Neutral Red (NR, B) (4, 15).

Thiosemicarbazones have had a lengthy history as potential prophylactic therapeutics for human disease beginning at least as early as 1946 (1). Thiosemicarbazones were the first antiviral compounds recognized to have a broad-spectrum antiviral activity against a range of DNA and RNA viruses. The use of N-methylisatin-*β*-thiosemicarbazone (methisazone/marboran) as an effective antiviral drug in the chemoprophylaxis of small pox was demonstrated in human volunteers in South India as early as 1965 (2, 3). Isatin-*β*-thiosemicarbazone (IBT, Figure 1 A) derivatives were found to demonstrate a range of antiviral activities (Moloney leukaemia virus, vaccinia virus), as reported by earlier studies (4, 5).

Isatin-*β*-thiosemicarbazone and some of its derivatives have high activity against neurovaecinia and certain other poxvirus infections in mice (6). The thiosemicarbazone of isatin was found strongly active (7). 

Isatin-*β*-thiosemicarbazone inhibits the formation of mature vaccinia virus progeny (8). Heterocyclic thiosemicarbazones exercise their beneficial therapeutic properties in mammalian cells by inhibiting ribonucleotide reductase, a key enzyme in the synthesis of DNA precursors (9). In another study isatin-3-thiosemicarbazone, revealed significant activity against MTB H37Rv (10). Thiosemicarbazone derivatives of isatin were tested for the inhibition of HIV nucleoprotein synthesis (11).

Isatin hydrazones form an interesting class of compounds which find wide spread applications in various fields including inhibition of DNA synthesis and induction of interferon secretion. However, the presence of an intramolecular hydrogen bond (H-bond) in these compounds allows one to expect that such bonds can also exist in the molecules of compounds. Then, the formation of the third (pseudo) cycle in these structures also makes them potential DNA intercalators. Thus, the isatin hydrazones are capable to intercalate into DNA base pairs (12). 

Based on the mentioned properties of hydrazones such as antibacterial, antifungal, anti-tubercular, antitumor activity, and many others, we report here the interaction between this hydrazone and calf thymus DNA (CT-DNA) using different instrumental methods. Our previous work showed that isatin can bind to CT-DNA via groove binding (13). In this study we show that the binding affinity of the IBT to DNA is much higher than that of isatin.

Neutral Red (NR, Figure 1 B) is a planar phenazine dye, and in general, is structurally similar to other planar dyes (14), *e.g*., those of the acridine, thiazine and xanthene kind. In recent years, the interaction of the fluorescent NR dye with DNA has been demonstrated by spectrophotometric (15) and electrochemical (16) techniques. Compared with a common ﬂuorimetric probe, ethidium bromide (EB), the NR dye offers lower toxicity, higher stability and convenience of use. In addition, its solution remains stable for up to 2 years. In this work, NR was selected as the probe (17).

Information obtained from this study will be helpful to understand the mechanism of isatin derivatives interaction with nucleic acids, and should be useful to develop potential probes of DNA structure and new therapeutic reagents for some diseases.

## Experimental

*Materials*

The highly polymerized CT-DNA and Tris–HCl were purchased from Sigma Co. Isatin and thiosemicarbazide were purchased from Merck. All solutions were prepared using double-distilled water. Neutral Red dye (Merck product) stock solution (1.0×10^−3^ molL^-1^) was prepared by dissolving its crystals in water and diluted to the required volume. Tris-HCl buffer solution was prepared from (tris-(hydroxymethyl)-amino-methane–hydrogen chloride) and pH was adjusted to 7.4. The stock solution of DNA-polymerized CT-DNA was prepared by dissolving DNA in 10 mM Tris-HCl buffer pH 7.4 and dialyzing exhaustively against the same buffer for 24 h. The DNA solution was stored at 4 ˚C and used within 5 days of preparation. CT-DNA solutions gave a ratio of UV absorbance at 260 and 280 nm more than 1.8, indicating that DNA was sufficiently free from protein (18). The concentration of the nucleotide was determined by UV absorption spectroscopy using the molar absorption coefficient (ε = 6600M^-1^cm^-1^) at 260 nm. An IBT stock solution (1×10^-3^ M) was prepared by dissolving an appropriate amount of compound in Tris-HCl buffer/DMSO (90:10%). It has been verified that the low DMSO percentage added to DNA solution does not interfere with the nucleic acid (19). 

*Synthesis and characterization of the isatin-β-thiosemicarbazone (IBT)*

IBT was obtained by refluxing isatin and thiosemicarbazide (1:1 molar ratio) in ethanol, following a general procedure (20). Powdered solid obtained after cooling were filtrated, washed with ethanol and dried under vacuum.

Yield: 68% obtained as yellow crystals; m.p.: 248-249 ºC; IR (KBr): ν_max_/cm^-1^ 3422 (NH_2_), 3238 (N–H), 1682 (C=O), 1593, 1539 (C=C, arom), 1342 (C=S); ^1^HNMR (200 MHz, DMSO-*d*_6_): 12.45 (1H, s, N-H), 11.18 (1H, s, N-H), 9.02 (1H, s), 8.67 (1H, s), 7.65 (1H, d, *J*_HH_ = 7.4 Hz), 7.35 (1H, t, *J*_HH_ = 14.7 Hz), 7.08 (1H, t, *J*_HH_ = 11.2 Hz), 6.91 (1H, d, *J*_HH_ = 7.8 Hz); Anal. Calcd for C_9_H_8_N_4_OS: C 49.1%, H 3.7%, N 25.5%. Found: C 48.5%, H 3.2%, N 25; *λ*_max_ (nm): 252, 271, 358.

*Instrumentation*

^1^H NMR spectra were recorded using a Bruker Avance DPX200MHz (4.7 Tesla) spectrometer with d_6_–DMSO as the solvent. The elemental analysis was performed using a Heraeus CHN elemental analyzer.

Absorbance spectra were recorded using an HP spectrophotometer (Agilent 8453) equipped with a thermostated bath (Huber polysat cc1). Absorption titration experiments were conducted by keeping the concentration of IBT constant (5×10^-5^ M) while varying the DNA concentration from 0 to 8×10^-5^M (ri = [DNA]/[IBT] = 0.0 –1.6).

Equal small aliquots of DNA stock solution were added to both IBT and reference solutions to eliminate the effect of DNA absorbance. Absorbance was recorded at 356 nm for IBT after DNA addition.

Fluorescence measurements were carried out with a JASCO spectrofluorimeter (FP 6200) by keeping the concentration of IBT constant (37µM) while varying the DNA concentration from 0 to 74 µM (ri = [DNA]/[IBT] = 0, 0.2, 0.5, 0.8, 1, 1.5, 1.8, and 2) .

The competitive interaction between the NR dye and the IBT with DNA in fluorescence measurements was carried out with the following setting: 541 nm as excitation wavelength, 618 nm as emission wavelength, 10 nm as excitation slit and 10 nm as emission slit (21). The experiment was carried out in 10 mM Tris–HCl at pH 7.4 in aqueous media. Constant amounts of the NR and DNA were titrated with increasing amounts of IBT solution. The changes in absorbance values or fluorescence intensities, as appropriate, were monitored against a blank after each addition of the IBT (17). 

Viscosity measurements were made using a viscometer (SCHOT AVS 450) which was maintained at 25 ± 0.5 ˚C using a constant temperature bath. The DNA concentration was fixed at 5×10^-5^ M, and flow time was measured with a digital stopwatch. The mean values of three replicated measurements were used to evaluate the viscosity (η) of the samples. The values for relative specific viscosity (η/η_0_)^1/3^, where η_0_ and η are the specific viscosity contributions of DNA in the absence (η_0_) and in the presence of the IBT were plotted against ri = [IBT]/[DNA] = 0.0, 0.5, 1.0, 1.2, 1.5, 1.8 and 2.0. 

CD measurements were recorded on a JASCO (J-810) spectropolarimeter by keeping the concentration of DNA constant (8×10^-5^ M) while varying the IINH concentration from 0 to 5.6×10^-5^M (ri = [IINH]/[DNA] = 0.0, 0.05, 0.4, and 0.7).

## Results and Discussion

DNA is a remarkable bioreceptor for a variety of small molecules and it can be considered as a major biological target to design anticancer agents. DNA-intercalating agents have been studied for several decades, and a few representatives (ellipticine, anthracyclines, acridines, and anthraquinones) are routinely used in the clinic for the treatment of cancers (22). This study shows how different methods, such as UV-visible, fluorescence, viscosity, and competitive interaction between the NR dye and IBT with DNA, can be used to elucidate the binding of IBT to target DNA molecules.

*Absorption spectra studies*

*Absorption spectra of IBT interaction with DNA*

**Figure 2:**
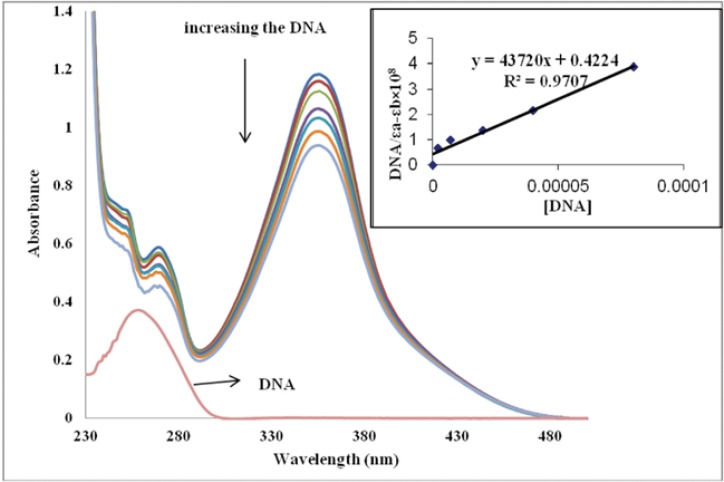
Absorption spectra of the IBT in 0.01 M Tris-HCl buffer (pH 7.4) at room temperature in the presence of increasing amounts of CT-DNA. [IBT] = 50 µM, [DNA] = 0–80 µM from top to the bottom. Arrows indicate the change in absorbance upon increasing the DNA concentration. Inset: plot of [DNA]/εa-εb vs. [DNA]

The electronic absorption spectroscopy is the most common way to investigate the interactions of compounds with DNA. A compound binding to DNA through intercalation usually results in hypochromism and bathochromism, due to the intercalation mode involving a strong π–π stacking interaction between an aromatic chromophore and the base pairs of DNA. It seems to be generally accepted that the extent of the hypochromism in the UV–visible band is consistent with the strength of intercalative interaction. The absorption spectra of IBT in the absence and presence of CT-DNA (at a constant concentration of IBT, [IBT] = 50 µM) are given in Figure 2.

Titration of calf thymus DNA with the IBT caused some spectral changes. The bands at 356 nm showed considerable hypochromism, up to 22% and a small shift of the absorption maximum (from 356 to 357 nm) when saturated around [DNA]/[IBT] = 1.6.

Large hypochromism in the absorption bands of the IBT in the presence of double helical DNA is usually characteristic of intercalation of molecule into DNA base pairs, due to the strong stacking interaction between the aromatic chromophore and the base pairs (23). So, the above phenomenon is indicative of most probable intercalative binding mode of IBT with calf thymus DNA (24, 25). It should be noted that no significant effect on the absorption bands of the molecule in the presence of double helical DNA, is characteristic of a groove binder. 

For further study of IBT interaction with DNA, the intrinsic binding constant between mentioned molecule and DNA was calculated; the intrinsic binding constant, K_b_ for IBT with CT-DNA was determined according to the following equation:

[DNA]/(ε_a_-ε_f_) = [DNA]/(ε_b_-ε_f_) + 1/K_b_(ε_b_-ε_f_)                      Equation(1)

Where [DNA] is the concentration of DNA in base pairs. ε_a_, ε_f_ and ε_b_ are the apparent, free and bound complex extinction coefficients, respectively. In particular, ε_f _was determined by a calibration curve of the isolated IBT in aqueous solution, following Beer’s law. ε_a_ was determined as the ratio between the measured absorbance and the IBT concentration, A_obs_/ [IBT]. A plot of [DNA]/(ε_a_-ε_f_) vs. [DNA] gives a slope of 1/( ε_b_-ε_f_) and a y-intercept equal to 1/K_b_ (ε_b_-ε_f_); K_b_ is the ratio of the slope to the y-intercept (Inset in Figure 2). The K_b_ value was calculated to be 1.03×10^5^ M^-1 ^(24). In the previous study (13), a K_b _value of 7.32×10^4 ^M^-1^ was determined (using UV) for isatin (a groove binder). The binding constants indicate that IBT binds more strongly than isatin. This result is expected, since IBT possesses greater planer area and extended π system than that of isatin, which will lead to IBT penetrating more deeply into, and stacking more strongly with base pairs of DNA (26). These data indicate that the intercalated drug has a significant effect on the strength of DNA binding.

*Absorption spectra of NR dye interaction with DNA*

In general, if a small molecule interacts with DNA, changes in absorbance (hypochromism) and in the position of the band (red shift) should occur. Hypochromism and red shift are important evidences indicating that the small molecule has intercalated between DNA base pairs, and is involved in a strong interaction in the molecular stack between the aromatic chromophore and the base pairs (27, 28). The spectral effects could be considered as follows: the empty π*-orbital of the small molecule couples with the π-orbital of the DNA base pairs, which causes an energy decrease, and a decrease of the π →π* transition energy. Therefore, the absorption of the small molecule should exhibit a red shift. At the same time, the empty π*-orbital is partially filled by electrons, reducing the transition probability, which leads to hypochromism.

In our work, UV–Vis spectra were recorded from solutions of the NR at constant concentration mixed with different concentrations of the DNA. In general, there is a maximum absorption at about 454 nm in the spectrum of an NR solution when DNA is present. It was found that the band absorbance at 454 nm decreased with an observed hypochromicity of 37%, and a small red shift (~11 nm) was evident with the increasing concentration of DNA. A band developed at approximately 500 nm with the increasing of DNA concentration, and has been designated to the NR-DNA complex. It increases in intensity with DNA, and has a bathochromic shift to 540 nm. An isosbestic point at 494 nm provided evidence of the new DNA–NR complex formation (29, 33).

*Absorption spectra of competitive interaction of IBT and NR with DNA*

**Figure 3:**
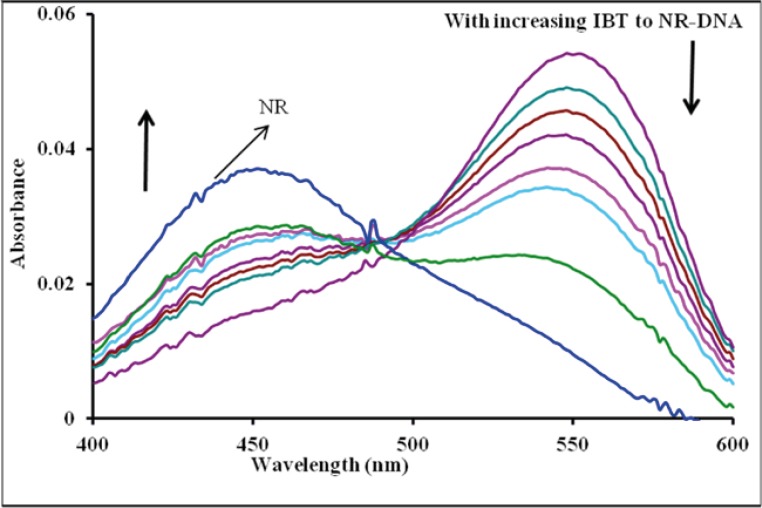
Effect of IBT on absorption spectra of NR-DNA: C_DNA_ = 8×10^−5^ M, C_NR_ = 2.00×10^−5^ M, and C_IBT_ = 0.0, 0.39, 1.5, 2.3, 3.4, 4.9, 7.5 and 11.5×10^−5^ M, corresponding to the curves, respectively

As shown in Figure 3, with increasing the concentration of IBT, the maximum absorption at 540 nm of the NR-DNA spectrum decreases but a slight intensity increase is observed in the developing band at 454 nm. Comparing this observation to the behavior of the free NR absorption band at 454 nm in the presence of increasing concentration of DNA, it is noted that its intensity increases. The observed changes in intensity and position of the bands with increasing amounts of IBT added to the NR-DNA complex solution suggest that some of the NR molecules, which were intercalated into the DNA base pairs, were exchanged by IBT. This is consistent with the view that planarity of a molecule is one of the necessary conditions for efficient intercalation into the double helix (30). An aromatic ring stacking between nucleobase pairs is regarded as a major driving force for binding of an intercalator into the double helix. Since the IBT molecule contains a planar aromatic ring (Figure 1), which can stack between DNA bases, IBT should be able to intercalate into the double helix. Thus, considering the above results, it is evident that with the use of NR as an indicator, and UV–Vis spectrophotometry to probe the interaction between IBT and DNA under neutral pH conditions, the drug, IBT, binds to DNA by intercalating between the base pairs (17, 29, 31).

*Fluorescence spectroscopic* *studies*

*Fluorescence spectra of IBT interaction with DNA*

**Figure 4:**
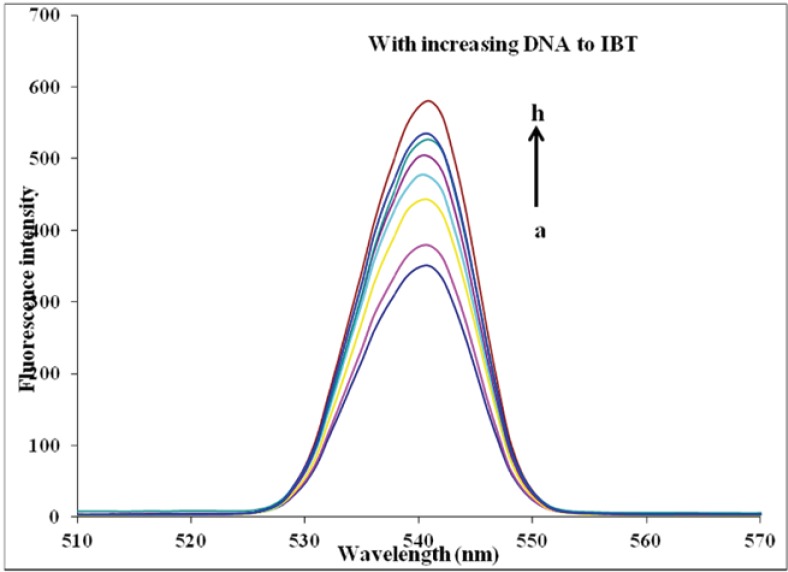
Emission enhancement spectra of IBT (37 µM) in the absence (bottom spectrum) and presence of increasing amounts of calf thymus DNA (7.4, 18.5, 29.6, 37, 55.5, 66.6 and 74 µM; subsequent spectra). Arrow shows the emission intensity changes upon increasing DNA concentration

The IBT can emit luminescence in Tris buffer with a maximum wavelength of about 541 nm. Figure 4 shows the emission spectrum of IBT in the absence and presence of varying amounts of DNA. As seen from the Figure, the intensity of emission at 541 nm increases appreciably in the presence of DNA. The enhancements of emission intensity imply that IBT molecules can insert between DNA base pairs. As a result, IBT molecules are protected from solvent water molecules by the hydrophobic environment inside the DNA helix; the accessibility of solvent water molecules to these compounds is reduced. The binding of IBT molecules to DNA leading to a marked increase in emission intensity also agrees with those observed for other intercalators (34, 35).

*Characteristics of fluorescence spectra of interaction between IBT and DNA–NR*

**Figure 5:**
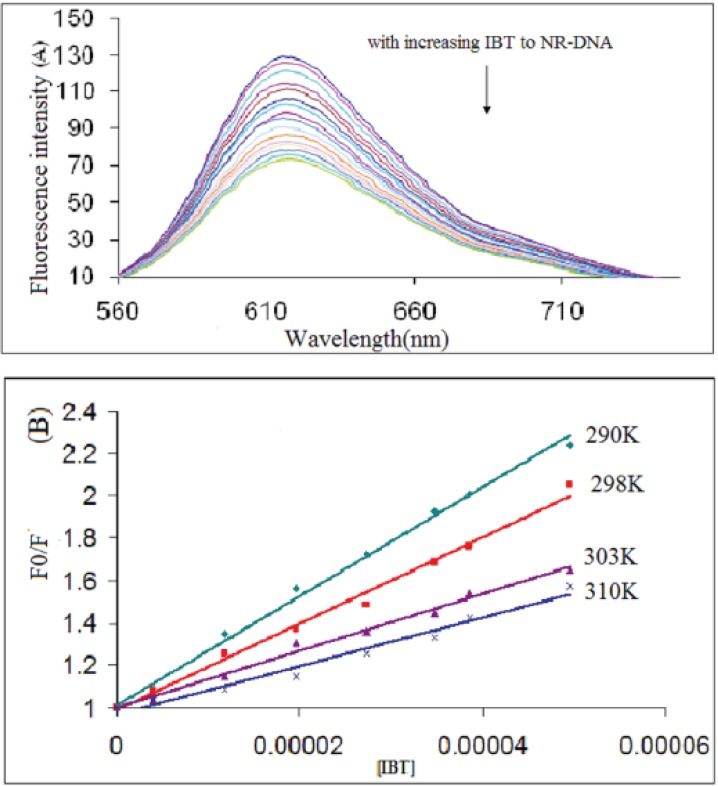
(A) Fluorescence emission spectra of the competition between IBT and NR. C_DNA_ = 9.0×10^−5^ M, C_NR_ = 3.0×10^−5^ M and IBT = 0 - 4.9×10^-5^ M corresponding to the curves, respectively. (B) The Stern–Volmer plots for the quenching of DNA–NR by IBT at different temperatures.

The interaction procedure of NR with CT-DNA at pH 7.4 was characterized by the fluorescence spectra (32, 33). They showed that initially, NR produced only weak fluorescence in the Tris–HCl buffer due to quenching by the solvent molecules. However, with the addition of increasing concentration of DNA, fluorescence intensity of NR was enhanced substantially because of its intercalation into DNA base pairs. Such spectral effects are similar to those observed in the interaction of ethidium bromide (EB) and DNA (28). This molecule is well known as a typical intercalator, and it has a weak fluorescence, which in the presence of DNA is significantly enhanced. This is attributed to strong intercalation of EB between the adjacent DNA base pairs (24). In the present study, with the addition of IBT to a solution DNA–NR, some NR molecules were released into solution after an exchange with the IBT, and this resulted in fluorescence quenching together (Figure 5A), indicating that interactions between DNA–NR and IBT occurred and DNA–IBT complex may form.

Fluorescence quenching refers to any process that is a decrease of the fluorescence intensity from a fluorophore due to a variety of molecular interactions. These include excited-state reactions, molecular rearrangements, energy transfer, ground-state complex formation, and collisional quenching. Quenching can occur by different mechanisms, which usually classified as dynamic quenching and static quenching. In general, dynamic and static quenching can be distinguished by their different dependence on temperature (36). Dynamic quenching depends upon diffusion. Since higher temperatures result in larger diffusion coefficients, the bimolecular quenching constants are expected to increase with increasing temperature. In contrast, increased temperature is likely to result in decreased stability of complexes, and thus lower values of the static quenching constants (19, 36-39)*.*

The data obtained was analyzed by the Stern–Volmer equation (Equation 2) (40):

 F_0_/F = 1 + K_q_ τ_0_ [Q] = 1 + K_sv _[Q]                    Equation (2) 

Where F_0_ and F are the ﬂuorescence intensities of DNA–NR in the absence and presence of the quencher, respectively. K_q _is the quenching rate constant of the biomolecule, K_SV_ is the Stern–Volmer quenching constant which can be considered as a measure for efficiency of fluorescence quenching by IBT, τ_0_ is the average lifetime of the biomolecule without quencher, and [Q] is the concentration of quencher (IBT). 

The K_sv _of NR-DNA fluorescence by IBT at different temperatures (290, 298, 303, and 310 K) was obtained and the results are shown in Figure 5B and indicated in Table 1. These results show that IBT can quench NR-DNA fluorescence in a static quenching procedure, because the K_sv_ has been decreased by temperature rising (33).

According to literature (44) τ ≈10^-8^ s, so the bimolecular quenching constant (K_q_) was calculated to be 1.14×10^12 ^at 310 K. It is larger than the limiting diffusion rate constant of biomolecule (2.00×10^10^), which indicates the static quenching occurred in the NR-DNA quenching by IBT (37, 41).

**Table 1:** The quenching constants of NR-DNA by IBT at different temperatures ranging from 0 to 4.9×10^-5 ^M, [DNA] = 9×10^-5 ^M, [NR] = 3×10^-5^ M, Binding constants (K_f_), number of binding sites (n) and relative thermodynamic parameters of IBT-DNA system.

Temperature (K)	K_sv_(L/mol)×10^4^	K_q_(L/mol)×10^12^	K_f_	n	ΔG (kJ/mol)	ΔH (kJ/mol)	ΔS (J/molK)
290	2.57	2.57	1.04×10^5^	1.13	-71.664	-49.87	-75.152
298	2.04	2.04	7.52×10^4^	1.13	-72.265		
303	1.34	1.34	4.77×10^4^	1.12	-72.641		
310	1.14	1.14	2.82×10^4^	1.10	-73.167		

*Binding constant and binding sites*

As summed small molecules bind independently to a set of equivalent sites on a macromolecule, the equilibrium between free and bound molecules is given by the equation (36, 37):

 log(F_0_ − F)/F = log K_f _+ n log[Q]                    Eqution (3)

Where K and n are the binding constant and the number of binding sites, respectively. According to Equation (3), the values of K_f_ and n can be obtained, which are summarized in Table 1. The results show that the IBT binds to DNA with high affinity, which is much higher than that of isatin interacting with DNA, suggesting that the biological activities of IBT are superior to isatin (13). The binding constant decreased with the rising temperature, which indicated that the binding is exothermic reaction.

The value of n approximately equal to 1 indicates the existence of just a single binding site in DNA for IBT, in other words the IBT molecules intercalate between all of the interbase spaces (33, 42).

*The thermodynamic parameters and nature of the binding forces*

Considering the dependence of binding constant on temperature, a thermodynamic process was considered to be responsible for the formation of a complex. Therefore, the thermodynamic parameters dependent on temperatures were analyzed in order to further characterize the interaction forces between IBT and DNA. The interaction forces between a small molecule and macromolecule include hydrogen bonds, van der Waals force, hydrophobic force, electrostatic interactions, *etc*. The thermodynamic parameters of binding reaction are the main evidence to confirm the binding force. If the enthalpy change (ΔH) does not vary significantly over the temperature range studied, then its value and that of entropy change (ΔS) can be determined from the van’t Hoff equation (43):

 log K = -ΔH/2.303RT + ΔS/2.303R                    Equation (4)

 ΔG = ΔH - T ΔS                    Equation (5)

Where K is the binding constantat corresponding temperature and R is the gas constant. The temperatures used were 290, 298, 303 and 310K. The enthalpy change (ΔH) and entropy change (ΔS) were obtained from the slope and intercept of the linear van’t Hoff plot based on log K versus 1/T . The free energy change (ΔG) was estimated from Equation (5). The values of ΔH, ΔS and ΔG are listed in Table 1. From Table 1, it can be seen that the negative value of ΔG revealed the interaction process is spontaneous. 

The free-energy changes (ΔG) for IBT–DNA interactions are negatively large due to their strong association. Ross and Subramanian reported that when ΔH<0 or ΔH≈0 and ΔS>0, the electrostatic force dominates the interaction; when ΔH<0 and ΔS<0, van der Waals interactions or hydrogen bonds dominate the reaction (44); when ΔH>0 and ΔS>0, hydrophobic interactions dominate the binding process. Results indicated that ΔH<0 and ΔS<0. Therefore, van der Waals interactions or hydrogen bonds are the main forces in the binding of the investigated drug to CT-DNA, and the mode of binding is intercalation. In addition, the negative entropy change results from the intercalation of IBT between CT-DNA bases, accompanied by the loss of translational and rotational degrees of freedom (45-47). It also should be added that groove binding is predominantly entropically driven, whereas intercalation is enthalpically driven (46). 

*Viscosity measurements*

**Figure 6:**
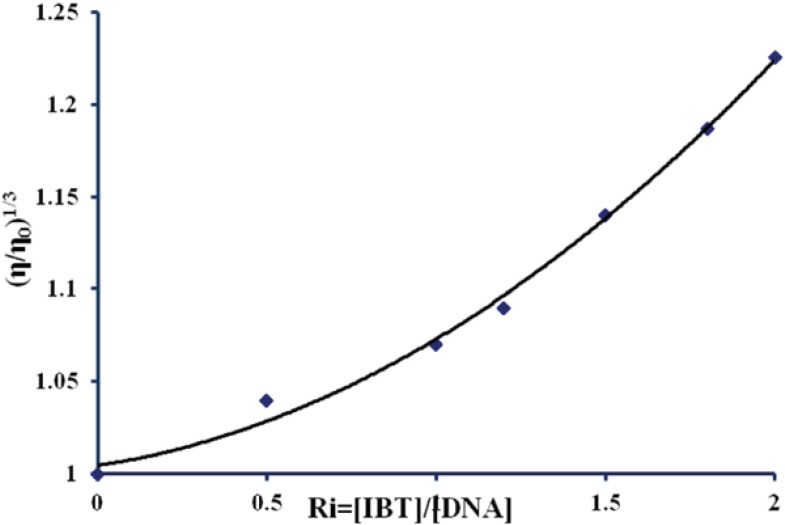
Effect of increasing amounts of IBT on the viscosity of calf thymus DNA (5× 10^ -5^M) in 10 mM Tris–HCl buffer (pH 7.4) at 298K (ri = [IBT]/[DNA] = 0.0, 0.5, 1, 1.2, 1.5, 1.8, and 2).

To further clarify the nature of the interaction between the IBT and DNA, viscosity measurements were carried out by varying the concentration of the added IBT to DNA solution. The specific viscosity of the DNA sample clearly increases with the addition of the IBT (Figure 6). The viscosity studies provide a strong evidence for intercalation. The viscosity increase of DNA is attributed to the intercalative binding mode of the IBT, due to effective DNA length increase (48, 49). 

A classical intercalative mode causes a significant increase in viscosity of DNA due to an increase in separation of base Pairs at intercalation sites, and hence an increase in overall DNA length happens. By contrast, complexes that bind exclusively with the DNA grooves by partial and/or non classical intercalation, under the same conditions, typically cause less positive or negative or no changes in the DNA solution viscosity, for example isatin causes no significant changes in the DNA viscosity (35, 50, 51).

*Circular dichroism spectral studies*

Circular dichroism (CD) spectroscopy is a very practical method to analyze the structure of optically active materials, such as DNA and proteins; therefore, the CD technique was used to determine the DNA conformational changes. In addition CD signals are quite sensitive to the mode of DNA interaction with small molecules.

**Figure 7:**
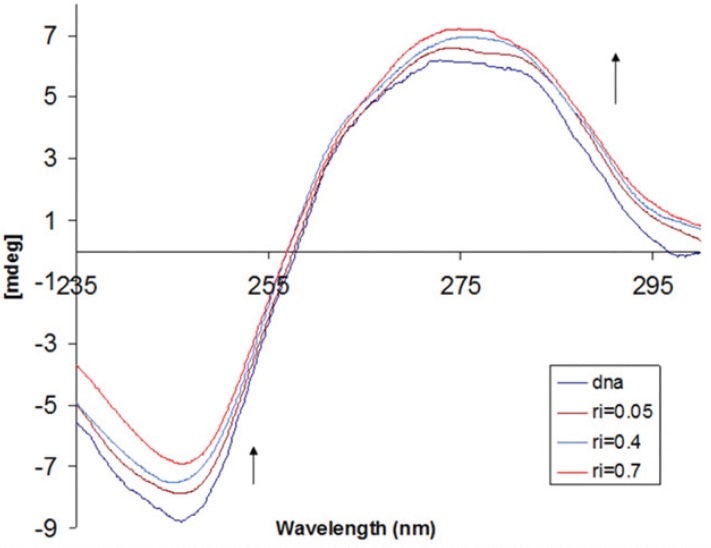
Circular dichroism spectra of DNA (8.0×10^-5^ M) in 10 mM Tris-HCl buffer, in the presence of increasing amounts of IBT (ri = [IBT]/[DNA] = 0.0, 0.05, 0.4, and 0.7).

The CT-DNA in the B conformation shows two conservative CD bands in the UV region; a positive band at around 275 nm (due to base stacking) and a negative band at around 245 nm (due to polynucleotide helicity) (52). In Figure 7, the changes in the CD spectrum of CT-DNA in presence of increasing concentrations of IBT are depicted. The positive band showed increase in molar ellipticity without any significant shift in the band maxima when the IBT concentration was progressively increased. This observation is supportive for the intercalative mode of binding of the IBT, where the IBT molecules stack in between the base pairs of DNA thus leading to the enhancement in the positive band (53). It is also evident from the CD spectrum that the binding of the IBT leads to decrement in helicity and consequently change in the conformation of CT-DNA, shifting from a more B-like to a more A-like DNA, which is indicative of intercalative binding mode of mentioned molecule to DNA molecule (44). 

## Conclusion

We examined the interaction of IBT to DNA and found sufficient evidences for its binding mode. We view this as a significant finding because of the ubiquitous medical roles found for IBT. The results obtained collectively show that IBT strongly interact with CT-DNA, by an intercalative mechanism. The interaction occurrence is supported by the following findings:

(i) The absorption spectrum of the IBT shows that as the concentration of DNA increases, a large degree of hypochromism develops in the spectrum. Hypochromism is usually arises from the strong stacking interaction between the aromatic chromophore and the base pairs. The intrinsic binding constant observed (1.03×10^5 ^M^-1^) was roughly comparable to other intercalators. 

(ii) The fluorescence studies showed an appreciable increase in IBT emission upon addition of DNA.

(iii) A competitive reaction monitored between Neutral Red (NR) dye, DNA and IBT showed that the intercalated NR was displaced from the DNA–NR system by IBT.

(iv) The positive slope in the Van’t Hoff plot indicates that the reaction between the IBT and DNA was enthalpy favored (∆H= - 49.87 kJ mol^-1^) and entropy disfavored (ΔS = -75.152 J mol^-1^ K).

(v) The viscosity increase of CT-DNA solution by adding the increasing amounts of IBT.

(vi) The obvious intensity increase in the positive band of CT-DNA in the CD spectrum.
